# The burden of hepatitis C in Europe from the patients’ perspective: a survey in 5 countries

**DOI:** 10.1186/1471-230X-13-16

**Published:** 2013-01-17

**Authors:** Jeffrey Vietri, Girish Prajapati, Antoine C El Khoury

**Affiliations:** 1 Health Outcomes Practice, Kantar Health, Independence Way Suite 220, Princeton, NJ, USA; 2 AllSource PPS, Long Beach, CA, USA; 3 Merck & Co., West Point, PA, USA

**Keywords:** Hepatitis C virus, Absenteeism, Presenteeism, Work impairment, Costs, Health status

## Abstract

**Background:**

Few studies have examined the impact of Hepatitis C virus (HCV) infection on patient reported outcomes in Europe. This study was conducted to assess the burden of HCV infection in terms of work productivity loss, activity impairment, health-related quality of life, healthcare resource utilization, and associated costs.

**Methods:**

The 2010 European National Health and Wellness Survey (n = 57,805) provided data. Patients reporting HCV infection in France, Germany, the UK, Italy, and Spain were matched to respondents without HCV using propensity scores. Outcome measures included the Work Productivity and Activity Impairment (WPAI) questionnaire and the Medical Outcomes Study Short Form-12 (SF-12v2) questionnaire. Subgroup analyses focused on treatment-naïve patients.

**Results:**

HCV Patients (*n* = 286) had more work impairment (30% vs. 18%, *p* < .001), more impairment in non-work activities (34% vs. 28%, *p* < .05), and more annual physician visits per patient (19.8 vs. 13.3, *p* < .001). Estimated indirect and direct costs were €2,956 (*p* < .01) and €495 (*p* < .001) higher than in matched controls, respectively. Health-related quality of life was also lower among HCV patients. Treatment-naïve HCV patients (*n* = 139) also reported higher work impairment (29% vs. 15%, *p* < .01), as well as more frequent physician visits (19.5 vs. 12.1, *p* < .01) than matched controls. Each treatment-naïve HCV infected patient incurred €934 in direct costs vs. €508 (*p* < .01 in matched controls. Employed treatment-naïve patients reported higher productivity loss per year compared to matched controls (€6,414 vs. €3,642, *p* < .05).

**Conclusion:**

HCV infection in Europe is associated with considerable economic and humanistic burden. This is also true of diagnosed patients who have never been treated for HCV.

## Background

An estimated 160 million people are chronically infected with Hepatitis C virus (HCV) worldwide [1]. Approximately 9 million have HCV infection in Europe, with greater prevalence in the southern and eastern regions [2-6]. While the incidence of new cases is low, few patients exposed to the virus spontaneously clear the infection, so exposure typically results in chronic infection that will continue indefinitely. Many chronically infected patients do not know that they have been infected with HCV, as infection is largely asymptomatic [7].

Though chronic HCV infection does not always cause serious health consequences, patients are at greater risk for development of cirrhosis, liver failure, and hepatocellular carcinoma (HCC), all of which are associated with high morbidity and mortality [8,9]. HCV infection is the most common indication for liver transplantation in Europe, and HCV infection of the transplanted liver is common. HCV was estimated to have caused more than 86,000 deaths in Europe in 2002 [4], a figure expected to increase as the patient population reaches the age at which long-term consequences of chronic infection typically manifest [4,10-12].

The goal of treatment for HCV is sustained virologic response (SVR), when the virus cannot be detected in the blood six months after the end of treatment. Patients who achieve SVR are at much lower risk for cirrhosis and HCC than those who do not achieve virological cure [13]. However, treatment with pegylated interferon and ribavirin is often unsuccessful, and is associated with adverse events and patient burden during the course of treatment [14-16]. Newly developed treatments for HCV infection are expected to dramatically increase the rate of SVR [17], and determining whether novel treatments are cost effective requires an assessment of the burden of HCV infection, including the economic impact as well as any impairment of quality of life.

Research using the US National Health and Wellness Survey (NHWS) has documented a significant burden of HCV infection on work productivity, with infected respondents missing approximately 9% of working hours in the last week, and reporting an average of 27% impairment while at work [18], and a database study found that HCV infected patients were 7.5% less productive based on work units per hour [19]. HCV is also associated with increased use of healthcare resources and increased direct healthcare costs [19-24]. The estimates of healthcare resource use vary greatly. DiBonventura et al. reported approximately 30% more physician visits among patients with diagnosed HCV infection than among propensity matched controls, with a similar trend for emergency room (ER) visits [24]. Database studies looking at costs in the year following diagnosis of HCV infection have found even larger differences. Davis et al. found that all-cause healthcare claims in HCV infected patients were, on average, almost US$21,000 in the year after diagnosis, nearly four times that in age, gender, and plan-matched controls [20], while McCombs et al. found an average cost of over US$37,000 in the year following diagnosis, which represents an incremental cost of more than US$23,000 [22]. Impaired health-related quality of life (HRQoL) in HCV population has also been documented [24-26]. However, almost all of these studies have focused on the US population, with very few studies describing the burden among HCV infected patients in Europe [26,27], and none from a patient perspective using a representative sample.

The objective of this study was to quantify the burden of HCV infection with respect to work productivity, healthcare resource use, related monetary cost to society, impairment in non-work activities, and HRQoL using a broadly representative sample of European adults.

## Methods

The National Health and Wellness Survey (NHWS; Kantar Health, New York, NY, USA), is an annual, cross-sectional survey of adults aged 18 years or older, with 57,805 respondents across France, Germany, Italy, Spain, and the UK in 2010. The NHWS is a self-administered survey which includes questions regarding diagnosed medical conditions, experience with over-the-counter and prescription medical treatments, health-related outcomes, as well as health-related attitudes and behaviors. Heath outcomes, including work productivity, impairment in activities of daily living, and HRQoL are assessed by validated scales (discussed below), and patients self-report the type and number of healthcare resources used during the six months preceding the survey. Potential respondents to the NHWS are recruited through an existing consumer panel selected by using opt-in emails, co-registration with panel partners, e-newsletter campaigns, and online banner placements. All panelists must explicitly agree to be a panel member, register with the panel through a unique email address, and complete an in-depth demographic registration profile. The sample is generated through stratified random sampling within this panel, with quotas based on gender and age to ensure the final sample is representative of each country’s adult (18 years of age and older) population. The 2010 NHWS was approved by Essex Institutional Review Board (Lebanon, NJ, USA), and all respondents provided informed consent.

For the present analysis, patients reporting Hepatitis B virus (HBV) infection, HIV, or AIDS were excluded to ensure that any burden documented in the HCV group was not due to HCV-associated comorbidities rather than the HCV itself. Likewise, because HCV infected patients are likely to differ from individuals without HCV in ways that may affect the outcomes measured by the survey, a propensity scoring methodology was employed to match each HCV infected patient with a single member of the control group [28]. Country of residence, age, gender, sexual orientation (heterosexual, homosexual, bisexual, or decline to answer), education (high school graduate vs. below), income (above or below median for country), number of non-liver comorbidities, smoking (yes/no), exercise (yes vs. no), alcohol use (yes vs. no), and BMI (underweight, normal weight, overweight, obese, or decline to answer) were included in a logistic regression to predict a self-reported physician diagnosis of HCV infection. The estimates resulting from this regression—the propensity scores—indicated how likely each HCV patient and non-HCV respondent was to have HCV given their demographic and health characteristics. The widely-used greedy matching algorithm was used to match each HCV patient to a single non-HCV control in the same country whose propensity score most closely matched the patent’s score [29].

The Work Productivity and Activity Impairment (WPAI) questionnaire was used to measure the impact of health on work performance [30]. The WPAI is a 6-item validated instrument that consists of four metrics: absenteeism (the percentage of work time missed because of one’s health), presenteeism (the percentage of impairment experienced while at work because of one’s health), overall work productivity loss (an overall impairment estimate that is a combination of absenteeism and presenteeism), and activity impairment (the percentage of impairment in daily activities because of one’s health). The recall period for all items is seven days. Only respondents who reported being employed full-time, employed part-time, or self-employed were shown the items assessing absenteeism, presenteeism, and overall work impairment, but all respondents provided data for activity impairment. Absenteeism was calculated by dividing the number of work hours a patient missed in the past week because of his or her health by the total number of hours a patient could have worked (the number of hours he/she did work plus the number of hours missed because of his/her health). Presenteeism was computed from patient’s rating of his or her level of impairment experienced while at work in the past 7 days on a scale from 0 (no impairment) to 10 (completely unable to function), which was then divided by 10 to create a percentage, with a range from 0% to 100%. Overall work impairment was measured by adding absenteeism and presenteeism to determine the total percentage of lost work time. Activity impairment was derived from patient’s rating of the level of impairment experienced in daily activities in the past 7 days on a scale from 0 (no impairment) to 10 (completely unable to function), which was then divided by 10 to create a percentage, with a range from 0 to 100%.

The 2010 NHWS also asked respondents about their use of healthcare resources over the preceding six months. Items included were the number of visits to healthcare providers and emergency room (ER), and hospitalization for the patient’s own medical condition. Healthcare providers include general practitioner/family practitioners, internists and dentists as well as more specialized physicians. The reported values were doubled to obtain an annual estimate.

We estimated direct healthcare costs for each NHWS respondent, and indirect costs for each employed respondent. Direct costs were estimated by multiplying the annualized healthcare resource use by the average cost of that service reported in the literature [31], then adjusting for inflation using the Harmonized Consumer Prices Index to 2010 values [32]. Indirect costs were estimated by projecting each patient’s absenteeism and presenteeism into an estimate of hours of lost productivity per year. Lost hours were then multiplied by an estimated hourly wage for each respondent. Hourly wages for all employed respondents (full-time, part-time, and self-employed) were calculated using the median wage of full-time workers using the most recently available (2006) personal income figures from Eurostat for full-time employees in each country [33]. These were inflated to 2010 values by inflating them according to the percentage rise in adjusted household income in each country from 2006 to 2010 as reported by Eurostat for the continental European countries, and by the rise in personal income from 2006–2010 for the UK as reported by the Office of National Statistics. The yearly wage was divided by the number of weeks typically worked each year to estimate the weekly value of the individual’s labor while at work. This weekly figure was then divided by the number of hours in a work week to create an hourly estimate. Figures for weeks and hours worked were obtained from the European Foundation for the Improvement of Living and Working Conditions [34].

The Medical Outcomes Study 12-Item Short Form Survey Instrument (SF-12v2) was used to assess HRQoL [35]. The instrument is designed to accurately report on eight health concepts (physical functioning, role physical, bodily pain, general health, vitality, social functioning, role emotional, and mental health) using the fewest questions possible. The SF-12 questions were selected from the widely used SF-36 health survey. The SF-12 allows calculation of physical component summary (PCS) and mental component summary (MCS) scores comparable to the ones calculated from SF-36. The present analysis included the PCS and MCS norm-based scores, which are scaled to have a mean of 50 and a standard deviation of 10 in the US population. Consequently, a 3 to 5-point difference is typically considered clinically significant [36-38]. In addition to generating summary PCS and MCS scores, the SF-12 can also be used to generate health state utilities using the SF-6D algorithm. The SF-6D is a preference-based single index measure for health using general population values [39]. The 18,000 health states that the SF-6D is able to describe are correlated with preference weights obtained from a sample of the UK general population using the recognized standard gamble valuation technique. The SF-6D index has interval scoring properties and yields summary scores on a theoretical 0–1 scale, where 0 is a state equivalent to death, and 1 is equivalent to perfect health. A .03 point difference is typically considered the minimally clinically important difference [40].

### Statistical analysis

Mean and standard deviation were calculated for all continuous measures, and frequencies and percentages were computed for categorical variables. Comparisons between HCV infected patients and controls (unmatched and matched) were made using chi-square tests for categorical outcomes and independent-samples t-tests for continuous outcomes. Because distributions of work impairment, activity impairment, and healthcare resource utilization were positively skewed, the Mann–Whitney U test was used in lieu of the *t*-test. An error rate of 5% was adopted for all hypothesis tests, which were conducted in SPSS version 19.0.

## Results

Of the 57,805 respondents in the 2010 European NHWS, 633 reported HBV, HIV, or AIDS, and were excluded from the sample. An additional 6 individuals were excluded because they reported HCV infection but indicated they had not been diagnosed by a physician. This resulted in a total sample size of 57,166, of which 286 reported a physician diagnosis of HCV. Of those reporting a diagnosis, 36 (12.6%) reported currently receiving prescription treatment for HCV, an additional 111 (38.8%) reported receiving prescription treatment in the past, and 250 (48.6%) reported never receiving prescription treatment. The 56,880 respondents who did not report ever experiencing HCV served as an unmatched control group.

As displayed in Table 1, HCV infected patients differed in a variety of ways from the non-HCV respondents in the European NHWS. Those with HCV infection were less likely to come from the UK or Germany, and more likely to come from Italy and Spain, consistent with the differences in HCV prevalence in those countries (all p < .05). HCV infected patients were older (53 vs. 46, p < .001), more likely to be male (58% vs. 48%, p < .001), or smoke cigarettes (46% vs. 28%, p < .001), and were less likely to decline to report their sexual orientation (2% vs. 4%, p < .001) or drink alcohol (68% vs. 79%, p < .001), although over two-thirds of the HCV infected patients reported doing so. HCV infected patients also had more co-morbid conditions, excluding liver disease (.69 vs. .27, p < .01).

**Table 1:** Demographic and health characteristics of the sample by HCV status

	**HCV group**		**Unmatched control group**		**p-value**
	**(n = 286)**		**(n = 56,880)**		
**Variable**	**n**	**%**	**n**	**%**	
Country					
France	66	23.1%	14835	26.1%	.248
Germany	60	21.0%	14859	26.1%	.048
UK	39	13.6%	14919	26.2%	<.001
Italy	83	29.0%	7358	12.9%	<.001
Spain	38	13.3%	4909	8.6%	.005
Female	119	41.6%	29352	51.6%	.001
Sexual orientation					
Heterosexual	253	88.5%	50937	89.6%	.548
Homosexual	11	3.8%	1306	2.3%	.081
Bi-sexual	17	5.9%	2311	4.1%	.108
Decline to answer	5	1.7%	2326	4.1%	.046
Married/living with partner	167	58.40%	36168	63.6%	.069
High school graduate	243	85.0%	46911	82.5%	.269
Income					
Above median	133	46.5%	25058	44.1%	.405
Below median	117	40.9%	23482	41.3%	.898
Decline to answer	36	12.6%	8340	14.7%	.322
BMI					
Underweight	8	2.8%	1590	2.8%	.998
Normal	107	37.4%	24002	42.4%	.102
Overweight	108	37.8%	18858	33.3%	.099
Obese	62	21.7%	10899	19.2%	.281
Decline to answer	1	0.4%	1531	2.7%	.014
Currently drink	194	67.8%	44797	78.8%	<.001
Currently exercise	153	53.5%	32373	56.9%	.244
Currently smoke	131	45.8%	16058	28.2%	<.001
	Mean	SD	Mean	SD	
Age	52.82	13.49	46.40	15.82	<.001
Number of co-morbid conditions	.69	1.04	.27	.67	.008

HCV patients generally reported worse outcomes than unmatched controls, presented in Table 2. HCV infected patients reported more impairment on all measures of work and activity impairment than unmatched control patients (all p < .001). Likewise, HCV patients also used more healthcare resources across all categories (all p < .01). The higher work productivity impairments and healthcare resource use observed among HCV patients resulted in higher estimated costs (all p < .01), presented in Table 3. HCV patients also had significantly worse HRQoL than non-HCV patients on all measures (all p < .001).

**Table 2:** Health outcomes among HCV patients and controls

	**HCV group**		**Unmatched controls**			**Matched controls**		
	**(n = 286)**		**(n = 56,880)**			**(n = 286)**		
	**Mean**	**SD**	**Mean**	**SD**	p-value*	**Mean**	**SD**	**p-value†**
Work impairment								
Absenteeism	7.78%	19.16%	5.44%	19.20%	<.001	5.45%	20.34%	.010
Presenteeism	26.27%	27.58%	15.83%	22.79%	<.001	14.45%	21.58%	<.001
Overall work impairment	30.45%	31.42%	19.39%	27.70%	<.001	18.30%	27.47%	<.001
Activity impairment	34.37%	30.60%	24.38%	28.16%	<.001	28.46%	30.57%	.011
Healthcare use								
Annual physician visits	19.80	23.93	10.66	14.64	<.001	13.26	19.21	<.001
Annual ER visits	0.68	2.11	0.38	1.90	.006	0.39	1.19	.330
Annual hospitalizations	0.52	1.59	0.26	2.14	<.001	0.27	0.98	.073
Quality of life (SF-12v2)								
MCS	44.17	9.85	46.58	10.59	<.001	46.36	10.79	.012
PCS	43.61	10.14	48.67	9.75	<.001	46.09	10.33	.004
Health utility	0.67	0.12	0.73	0.13	<.001	0.71	0.15	.001

Comparisons of work impairment include only the employed subsamples: 138 HCV patients, 32,179 non-HCV, and 134 matched controls.

*Indicates the significance comparing the HCV group with the unmatched control group.

†Indicates the significance comparing the HCV group with the matched control group.

**Table 3:** Costs among HCV patients and controls

	**HCV Patients**		**Non-HCV**			**Matched controls**		
	**(n = 286)**		**(n = 56,880)**			**(n = 286)**		
	**Mean**	**SD**	**Mean**	**SD**	p-value*	**Mean**	**SD**	**p-value**^**†**^
Indirect costs								
Absenteeism	€ 2,038.63	€ 6,048.12	€ 1,357.90	€ 5,326.81	<.001	€ 1,253.43	€ 4,884.92	.011
Presenteeism	€ 5,493.91	€ 6,898.97	€ 3,368.88	€ 5,479.10	<.001	€ 3,298.06	€ 5,454.64	.001
Total indirect	€ 7,532.54	€ 9,879.57	€ 4,729.20	€ 7,786.24	<.001	€ 4,576.29	€ 7,365.19	.002
Direct costs								
Physician visits	€ 486.61	€ 649.61	€ 288.54	€ 410.89	<.001	€ 329.52	€ 536.32	<.001
ER visits	€ 71.68	€ 233.24	€ 40.72	€ 207.97	.007	€ 42.94	€ 137.86	.360
Hospitalizations	€ 588.77	€ 1,867.19	€ 288.19	€ 2,754.17	<.001	€ 279.62	€ 991.20	.075
Total direct costs	€ 1,147.06	€ 2,265.46	€ 617.45	€ 2,889.90	<.001	€ 652.07	€ 1,373.94	<.001
Total costs	€ 4,587.54	€ 8,653.64	€ 3,291.01	€ 7,154.33	<.001	€ 2,685.28	€ 5,530.30	<.001

Comparisons indirect costs include only the employed subsamples: 138 HCV patients, 32,179 non-HCV, and 134 matched controls. Comparisons of direct and total costs include the full samples. Unemployed respondents’ indirect costs are included in total costs as €0.

*Indicates the significance comparing the HCV group with the unmatched control group. †Indicates the significance comparing the HCV group with the matched control group.

There were no significant differences on demographics between HCV infected patients and propensity matched controls (data not presented), though many of the decrements in outcomes associated with HCV remained significant (Table 2). Among HCV infected patients who reported being employed, health problems resulted in missing 43% more work than controls (7.8% vs. 5.5%, p < .05), and 80% greater impairment while at work (26.3% vs. 14.5%, p < .001), leading to 66% greater overall work impairment (30.5% vs. 18.3%, p < .001). Likewise, HCV was associated with greater impairment in non-work activities (34.4% vs. 28.5%, p < .05).

HCV patients also had more physician visits per year than matched controls (19.8 vs.13.3, p < .001). However, lack of power prevented the detection of differences between HCV patients and controls for annual ER visits (.68 vs. .39, p = .33) and hospitalizations (.52 vs. .27, p = .07), though these differences were of a similar magnitude as the significant differences between HCV patients and the unmatched non-HCV sample.

Costs were also estimated to be higher among HCV patients (Table 3). Employed HCV patients lost an average of €1,914 worth of productivity to absenteeism per year, 60% more than their matched controls (€1,195, p < .05). Impairment while at work (presenteeism) is also significantly more costly in HCV patients than controls, with annual productivity losses averaging €5,268 and €3,154 (p < .05), respectively, for an overall indirect cost of €7,182 per year, €2,810 more per employed patient per year than controls (p < .01 for the difference). Direct costs were also significantly elevated among HCV patients, with annual costs 76% greater than matched controls (€1,147 vs. €652, p < .001), driven by significantly greater costs due to physician visits (€487 vs. €330, p < .001) and hospitalization, though lack of power prevented the difference in hospitalization costs from reaching statistical significance (€589 vs. €280, p < .08).

HCV infection also impacted the three measures of HRQoL. HCV patients had a clinically meaningful .04 decrement in health utilities compared to matched controls (.67 vs. .71, p < .01). MCS scores in HCV were about 2 points lower than matched controls (44.17 vs. 46.36, p < .05), while PCS scores were about 2.5 points lower in HCV (43.61 vs. 46.36, p < .01), though these differences are slightly smaller than would be considered clinically meaningful.

### Treatment-naïve patients

Worse outcomes were also seen among the treatment-naïve subgroup (n = 139) relative to matched controls. Employed treatment-naïve patients reported significantly greater work impairment than matched controls. As displayed in Table 4, untreated HCV infection was associated with nearly twice the impairment while at work (presenteeism; 24.8% vs. 12.9%, p < .01). Overall work impairment was elevated to a similar degree (28.7% vs. 14.8%, p < .01). However, the difference in absenteeism did not reach statistical significance (6.7% vs. 2.4%, p = .16). Impairment in non-work activities was also similar across HCV status (31.7% vs. 29.3%, p = .38).

**Table 4:** Health outcomes in treatment-naïve HCV patients and matched controls

	**HCV never treated**		**Matched controls**		**p-value**
	**(n = 139)**		**(n = 139)**		
	**M**	**SD**	**M**	**SD**	
Work productivity & activity impairment					
Absenteeism	6.67%	18.26%	2.84%	13.36%	.157
Presenteeism	24.84%	30.07%	12.86%	21.28%	.006
Overall work impairment	28.65%	32.94%	14.75%	24.57%	.004
Activity impairment	31.73%	30.57%	29.28%	31.48%	.374
Healthcare resource utilization					
Physician visits	19.54	24.62	12.06	16.80	.001
ER visits	0.46	1.90	0.35	1.15	.986
Hospitalizations	0.36	1.16	0.19	0.99	.120
Quality of life (SF-12v2)					
MCS	44.72	10.43	45.93	11.64	.361
PCS	43.49	10.26	45.99	10.72	.048
Health utility	0.68	0.13	0.71	0.15	.080

Comparisons of work productivity impairment are based on 65 treatment-naïve HCV patients and 65 matched controls. All other comparisons include the entire samples.

Consistent with the trend observed in the larger HCV sample, treatment-naïve patients also used more healthcare resources than matched controls. HCV patients used all forms of healthcare at least as much as matched controls. Significantly, patients had about 60% more physician visits than controls (19.5 vs. 12.1, p < .01). Differences in ER visits (.45 vs. .35, p = .97) and hospitalizations (.36 vs. .19, p = .12) were not statistically significant.

The greater work impairment among employed treatment-naïve HCV patients is associated with significantly higher economic costs due to lost productivity. Table 5 shows the estimated value of the lost productivity. The incremental cost of HCV infection is €2,773 per employed patient per year, primarily because of greater presenteeism than matched controls. Likewise, more frequent healthcare use is reflected in higher estimated direct medical costs, which were greater to a significant degree for physician visits and total direct costs, incurring €427 more per year on average than matched controls. Overall, each treatment-naïve HCV patient was associated with an average overall incremental cost of €1,715 above the matched controls per year.

**Table 5:** Estimated costs in treatment-naïve HCV patients and matched controls

	**HCV Never treated**		**Matched controls**		**p-value**
	**(n = 139)**		**(n = 139)**		
	**M**	**SD**	**M**	**SD**	
Indirect costs					
Absenteeism	€ 1,293.61	€ 4,398.13	€ 579.62	€ 2,504.32	.204
Presenteeism	€ 5,120.80	€ 7,680.29	€ 3,062.29	€ 5,868.47	.015
Total indirect	€ 6,414.42	€ 8,587.96	€ 3,641.91	€ 6,699.54	.011
Direct costs					
Physician visits	€ 488.06	€ 720.02	€ 288.44	€ 458.58	.003
ER visits	€ 47.18	€ 178.64	€ 39.19	€ 140.43	.979
Hospitalizations	€ 399.05	€ 1,368.76	€ 179.88	€ 858.29	.124
Total direct	€ 934.30	€ 1,873.33	€ 507.50	€ 1,053.43	.005
Total costs	€ 3,834.44	€ 6,991.25	€ 2,119.56	€ 4,974.79	<.001

Indirect costs apply only to the 65 HCV patients and 65 matched controls who report being employed. Direct and total costs apply to the entire sample. Unemployed respondents’ indirect costs are included in total costs as €0.

Treatment-naïve patients also reported worse mean physical quality of life than matched controls (43.49 vs. 45.99, p < .05), though the magnitude of this effect is slightly below the level considered clinically meaningful. There was also a trend for patients to have lower health utilities (.68 vs. 71, p = .08), which would be considered clinically meaningful. The decrement on MCS scores associated with untreated HCV did not reach statistical significance (44.72 vs. 45.93, p = .36).

## Discussion

The present study included data from a large survey of European adults with and without HCV infection across five counties, which measured outcomes through widely used validated scales. Patients reporting a physician diagnosis of HCV infection had significantly impaired work productivity, greater impairment in non-work activities, more healthcare resource utilization, and worse HRQoL than both the general population without HCV and propensity-matched individuals without HCV infection. The economic costs of HCV infection are considerable. We estimated work-productivity impairment due to HCV costs over €7,500 per employed patient per year, an incremental indirect cost of almost €3,000 over matched controls. Direct costs are also elevated by almost €500 per patient when compared to matched controls. The intangible cost of lower HRQoL observed in this sample was also significant, particularly regarding physical quality of life and health utility. HCV patients had consistently worse outcomes than matched controls across almost all outcome measures, though some measures of healthcare resource use did not reach significance. This seems to be primarily an issue of statistical power, as those that did not reach significance—ER visits and hospitalizations—were those measuring rare events experienced by a minority of respondents. A previous study using the same measures but using a larger, US sample found significant results, despite observing numerically smaller differences between HCV patients and controls [24]. Otherwise, the HRQoL decrements and work impairment observed in the present analysis are consistent with those measured in the US NHWS, and of a similar magnitude [18].

In addition to examining HCV infected patients as a whole, the present study also considered treatment-naïve patients separately. The treatment-naïve patients are an especially interesting subgroup, as they are neither burdened by the adverse events of treatment, nor benefited from a successful therapy, and so may better represent the burden of untreated HCV infection. Relative to matched controls, these patients reported greater impairment at work and more frequent physician visits, and estimated costs were also higher. Unlike treatment-experienced patients, these individuals’ elevated resource use would not be due to treatment or adverse events associated with such treatment, nor would work impairments or reduced HRQoL be due to side effects. The pattern of results in this subgroup was generally consistent with the findings in the broader comparisons, with few exceptions. Treatment-naïve patients did not show significantly elevated absenteeism relative to matched controls, but the magnitude of the difference was actually larger than in the broader comparison, suggesting that this difference is simply due to a lack of power rather than a different pattern among treatment-naïve patients.

The use of propensity scoring matching ensures that none of the effects observed could be attributed to any demographic or health history variables included in the matching analyses. However, we cannot rule out the possibility that additional variables (such as prior drug use) not included in the matching may explain the observed differences in health outcomes. However, most of the likely factors (co-morbid health conditions, health behaviors, etc.) were equated by the matching procedure.

The self-report survey methodology did not allow us to verify HCV diagnosis. However, the findings coincide with those of previous studies, suggesting this HCV sample is similar to that of other, clinically-verified HCV samples. We were also unable to confirm that controls were free of HCV infection, and given that many HCV patients are unaware of the infection it is possible that HCV patients were in the control group, causing us to underestimate the impact of HCV infection. The current study did not assess reasons for healthcare resource utilization but, given the propensity score methodology, the assumption was made that the additional resources used by the HCV group were due to the virus itself, as none of the assessed demographic or non-HCV health history variables differed between the groups. Some selection bias may also be present, in that individuals who completed the survey may have differed in some meaningful way from those who did not respond. However, for such a bias to affect the conclusions, the effect of HCV would have to be different among those who chose not to complete the survey than in those who did respond, which seems unlikely. Finally, the modest number of HCV infected respondents limits the precision of the estimates of the associated burden, though this would not contribute to spurious positive findings.

## Conclusions

The humanistic and economic burden of HCV in Europe is substantial. The pattern of results is similar among treatment-naïve patients, which suggests that the burden of treatment is not driving the elevated resource use and lower HRQoL observed in the broader sample. Effective treatment of HCV may alleviate the work impairment associated with HCV and lower use of healthcare resources, while providing improved quality of life to the individual.

## Competing interests

Merck & Co. funded the analysis for this study, and markets a treatment for hepatitis C infection. JV is an employee of Kantar Health, which conducted the study with funding from Merck & Co. ACEK was an employee of Merck & Co. at the time the study was completed, and GP is a contractor to Merck & Co.

## Authors’ contributions

JV and ACEK conceived the study. JV conducted the data analysis. All authors reviewed the results and contributed to the writing of the final manuscript.

## Pre-publication history

The pre-publication history for this paper can be accessed here:

http://www.biomedcentral.com/1471-230X/13/16/prepub
